# The impact of exercise interventions concerning executive functions of children and adolescents with attention-deficit/hyperactive disorder: a systematic review and meta-analysis

**DOI:** 10.1186/s12966-021-01135-6

**Published:** 2021-05-22

**Authors:** Xiao Liang, Ru Li, Stephen H. S. Wong, Raymond K. W. Sum, Cindy H. P. Sit

**Affiliations:** 1 Department of Sports Science and Physical Education, The Chines University of Hong Kong, Shatin, New Territories Hong Kong, China; 2 Faculty of Physical Education, Shenzhen University, Shenzhen, China

**Keywords:** Physical activity, Executive functions, Children and adolescents, ADHD

## Abstract

**Background:**

Previous studies found that exercise interventions have positive effects on executive functions of the general population. However, studies seldom target executive functions of children and adolescents with attention-deficit hyperactivity disorder (ADHD). This study aimed to synthesise empirical studies regarding the effects of exercise interventions on executive functions of children and adolescents with ADHD.

**Methods:**

A systematic search of the relevant literature was conducted in March 2020 through six electronic databases: CINAHL Complete, Eric, MEDLINE, PsychINFO, SPORTDiscus with Full Text, and Web of Science. Randomised controlled trials/quasi-experimental designs that applied exercise interventions and assessed executive functions through neurocognitive tasks among children and adolescents with ADHD were included. Altogether, 314 studies were identified, from which 31 full texts were independently assessed by two authors for eligibility. Finally, 21 studies underwent systematic reviews and 15 were selected for meta-analysis. Data extraction procedures and risk of bias analysis were conducted by two independent authors using the Physiotherapy Evidence Database (PEDro) scale.

**Results:**

The findings indicated that exercise interventions improved overall executive functions of children and adolescents with ADHD (*SMD* = 0.611, 95% CI [0.386 to 0.836], *p* < 0.01). Exercise interventions had a moderate-to-large positive effect on inhibitory control (*g* = 0.761, 95% CI [0.376 to 1.146], *p* < 0.01) and cognitive flexibility (*g* = 0.780, 95% CI [0.331 to 1.228], *p* < 0.001). Likewise, during the subgroup analysis, intervention intensity and sessions of exercise (acute vs chronic) significantly moderated exercise intervention rather than intervention type.

**Conclusions:**

Chronic sessions of exercise interventions with moderate intensity should be incorporated as treatment for children with ADHD to promote executive functions.

**Supplementary Information:**

The online version contains supplementary material available at 10.1186/s12966-021-01135-6.

## Introduction

Attention-deficit/hyperactivity disorder (ADHD) is a neurodevelopmental condition commonly diagnosed during childhood [1]. The global prevalence of ADHD is around 5.29% [2], and up to 7.2% of those affected are children and adolescents [3]. Primarily, ADHD is associated with age-inappropriate behaviours, including hyperactivity, impulsivity, and inattention [4]. Executive dysfunction is an endophenotype of ADHD symptoms [5], yielding co-occurring medical and psychiatric illnesses [6], including behavioural sleep problems [7], physical inactivity [8], motor abnormalities, and impairments [9]. If individuals with ADHD do not receive effective interventions during childhood, ADHD symptoms and impairments will span into adulthood [10]. Further, the social and economic costs of untreated ADHD symptoms are considerable, including academic underachievement, unemployment, delinquency and higher rates of divorce than the general population [11, 12]. Therefore, improving executive dysfunctions among children and adolescents with ADHD is critical.

Executive functions (EFs) represent a set of cognitive skills that involve top-down control processes elicited in the planning, organising, and monitoring of complex, goal-directed behaviours [13]. EFs govern three core functions (inhibitory control, working memory, and cognitive flexibility) together with higher-level functions (reasoning, planning, and problem-solving) [14–16]. EF skills are necessary for children and adolescents’ social development [14], sleep duration and quality [17, 18], and physical and mental health [19]. Additionally, EFs are higher-order cognitive functions [20] facilitate successful academic learning, control stress-related activities, and suppress inappropriate behaviours among children with ADHD [21]. Inhibitory control and working memory have been reported as the most consistently impaired domains in ADHD [22]. Impaired inhibitory control in children with ADHD is closely associated with delayed self-regulation of emotions, deficits in self-directed speech and reduced allocation of attentional resources [23–25]. ADHD-related working memory deficits strongly predict visual inattention, social impairment including peer relationships and inattentive and hyperactive behaviours [26, 27]. Abnormal cognitive flexibility has been observed in individuals with ADHD and reflects inefficient and unsuccessful problem-solving skills and low academic achievements [28, 29].

Exercise is a behavioural treatment for children and adolescents with ADHD and has been used to treat ADHD symptoms such as, cognition, motor performance and social behaviours [30–33]. Preliminary results shows that both acute and chronic exercise are beneficial for cognition in children and adolescents with ADHD [28, 34]. Robust evidence concludes that exercise is a viable complementary method that positively affects cognitive function from early childhood [35] to adulthood [36] in the general population. Furthermore, it can diminish the risk of age-related cognitive decline [37]. Previous reviews validate that enhanced cognitive functioning due to exercise is evident in EFs among children and adolescents [38]. Additionally, prior reviews indicate that any type of exercise can promote cognitive performance [39]. For example, Best [40] stated that different types and levels of exercise could improve the EFs of children. This enhancement materialises in three ways: (1) through the cognitive demands inherent in the engagement of goal-directed activity, (2) through the cognitive engagement required in the participation of complex motor tasks, and (3) through the involvement of the brain in physiological changes during participation in aerobic exercise (AE). In addition, previous studies also found that the higher levels of exercise intensity were associated with improvement of cerebral oxygenation and blood volume in the brain, which resulted in improved prefrontal-dependent cognitive performance [41, 42].

Numerous scholars have begun to contest the well-established view that AE facilitates EFs. Diamond and Ling [43, 44] specified that aerobic or resistance training interventions were the least beneficial for the progress of EFs. Similarly, Takacs and Kassai [45] claimed that aerobic activity was insufficient as a cognitive engagement exercise, which requires the allocation of attentional resources and proficiency in various sports, such as yoga and ball games, to improve EFs [46]. Yet, the research of Diamond and Ling [43] was derived from seven studies that focused on a wide age group, and only three focused on children. Furthermore, Takacs and Kassai’s [45] review accentuated the efficacy of exercise interventions and comprised children with typical development and diverse clinical diagnoses. Therefore, it is unclear whether children can gain greater advantage from cognitively engaging exercises (CEE) (i.e., ball games, yoga, exergaming) or AE (i.e., swimming, running, jumping).

Many studies concur that exercise interventions mostly have positive effects (i.e., Hedges’ g or Cohen’s d) on EFs of the general population, including acute exercises for preadolescent children (g = 0.20 [− 0.04 to 0.42]) [20], adolescents (d = 0.52 [0.26 to 0.77]) [38], and older adults (g = 0.67 [0.40 to 0.93]) [47], together with chronic exercises for children and adolescents (d = 0.20 [0.09 to 0.30]) [16]. Nonetheless, only a few studies have reported the effects of exercise interventions on cognitive functioning and EFs, particularly among children and adolescents with ADHD [48].

We determined five gaps in previous literatures. First, most existing EF reviews focused on the general population, and only a handful of studies inspected individuals with executive dysfunctions. Research regarding children and adolescents with ADHD was even more scarce [37]. Second, only one meta-analysis [48] stressed upon the cognition of individuals with ADHD, stating that exercise interventions had a minimal-to-moderate effect (r = 0.181) on overall EFs among such individuals. Regardless, the interpretation of these results may have a high risk of bias, as the meta-analysis included one study [49] that focused on participants over the age of 18 years (i.e., college students with ADHD). Thus, it is difficult to interpret the effects of exercise interventions on EFs of children and adolescents with ADHD before young adulthood. Third, few reviews have distinguished between core EFs (i.e., cognitive flexibility, inhibitory control and working memory) and higher-level EFs (i.e., planning, reasoning and problem-solving), categorising the exercise intervention type into CEE and AE [16, 45]. Consequently, it is difficult to identify the specific type of exercise that contributes to a specific EF domain. Fourth, Diamond and Ling [43] affirmed that prolonged intervention durations generated better EF outcomes but required further confirmation regarding cognitive training in exercise interventions. Finally, current literature rarely differentiates between acute exercise interventions and chronic exercise interventions [48]; however, acute exercise could easily lead to improved effects on cognitive performance, but the benefits are temporary. Further, the positive effects of acute interventions are not associated with the positive effects of chronic interventions. Hence, it is imperative to determine the impact of acute and chronic exercises on EFs.

To the best of our knowledge, reviews seldom target EFs of children and adolescents with ADHD. Further, the core EFs (cognitive flexibility, inhibitory control, and working memory) are rarely discerned in the subgroup analysis to examine the effects of exercise interventions on domain-specific EFs of these individuals. Thus, in response to these gaps in the literature, the purpose of this systematic review was to synthesise published studies focusing on exercise interventions targeting on EFs for children and adolescents with ADHD.

## Methods

### Definitions

The meta-analysis amalgamated all available evidence regarding exercise interventions to facilitate EF skills among children and adolescents with ADHD. Primarily based on relevant reviews, we classified the EF outcome measures for the primary studies into three core-specific EF domains [14, 45]. Specifically, the modified flanker task was utilised to appraise both inhibitory control and cognitive flexibility [50].

### Search strategy

A systematic search was conducted in March 2020 through six electronic databases (inception to March 2020): CINAHL Complete, Eric, MEDLINE, PsychINFO, SPORTDiscus with Full Text, and Web of Science. The search was updated on February 2021 before submission. The search was limited to English, human-related, and peer-reviewed articles. The initial search was undertaken using four key terms: physical activity, executive function, ADHD, and children or adolescents. The search keywords for each main term were developed from the search strategies of previous reviews, in tandem with expert opinions in the fields of exercise interventions and EFs [16, 20, 48]. Additionally, two independent reviewers performed a manual search to select relevant articles from the previous systematic reviews [30, 48].

#### Inclusion and exclusion criteria

Studies were included if they:

1. Examined the effects of exercise interventions on EFs
2. Comprised participants with ADHD with a diagnosis by clinical or parent report aged between 5 and 18 years
3. Were based on intervention research (i.e., clinical and field trials)
4. Reported the results of outcome measures that used neurocognitive tasks (i.e., Stroop Task, Tower of London and Trail Making Task) of EFs
5. Were peer-reviewed articles available in full text and written in English

Studies were excluded if they:

1. Were written in a language other than English
2. Were based on observational research (i.e., cross-sectional, case-control, and cohort)
3. Included participants with other types of disabilities or the data specific to children and adolescents with ADHD could not be determined
4. Were interventions that did not involve exercise training
5. Were review studies, case/government reports, conference papers, book chapters, or policy documents

### Data selection

**Fig. 1:**
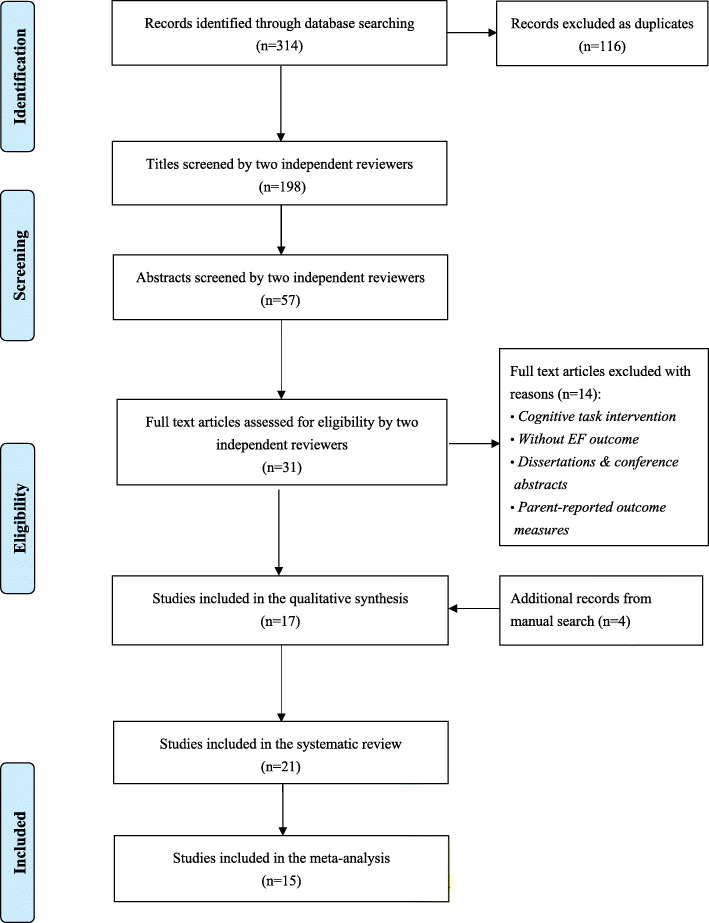
PRISMA flow study selection diagram

Inclusively, 314 studies were identified in the initial six-database search and no new articles were identified during the updated search in March 2021. Figure 1 illustrates the number of studies screened and those that met the inclusion criteria. To ensure the accuracy of the systematic search process, two reviewers familiar with EFs and exercise research administered the multi-step search process and screened the titles, abstracts, and full-length texts. The reviewers then independently made their initial assessments. The inter-rater reliability (k value) for reviewers’ abstract and full text screening was calculated (i.e., fair [0.40–0.59], good [0.60–0.74], and excellent agreement [> 0.75]) [51]. Upon any disagreement, a third reviewer was included to deliberate and make a final decision. Altogether, 57 abstracts met the inclusion criteria, with an inter-rater reliability of k = 0.78 between the two reviewers. Thereafter, 31 studies were selected for full text screening, and 17 studies passed the inclusion criteria with an inter-rater reliability of k = 0.87. Additionally, four manually searched studies were approved by the two reviewers as they met the inclusion criteria. Finally, 21 studies were selected for the systematic review, and 15 studies were included in the meta-analysis.

### Data extraction

Data were extracted using a standardised form that collected the following details: (a) descriptive information, such as author(s), year of publication, the country or region where the data were collected; (b) research design, including RCTs or non-randomised comparison studies (NRS) and crossover; (c) sample characteristics (i.e., participants’ age range, gender, clinical diagnosis method, and the sample size, including the number of participants in the intervention and control groups); (d) intervention characteristics (i.e., design, setting, frequency, intensity, length); (e) outcome measures (i.e., working memory, inhibitory control, cognitive flexibility); and (f) major findings.

### Meta-analytic procedures

The meta-analysis was implemented using Comprehensive Meta-Analysis (version 2.0). Only studies that reported sufficient statistical data from the pre- and post-test were included. The statistical analysis contained: (1) mean (M), sample size (N), and standard deviation (SD), which were the primary methods for effect size calculations; (2) when studies used two or more measuring tasks to assess the same EF domain, the one most frequently used task was included in the meta-analysis [16]; (3) for studies denoting multiple raw scores for one EF variable, the outcome of the more executive demanding condition was chosen (e.g., non-perseverative errors in the Wisconsin Card Sorting Test (WCST)); (4) when studies measured EF domains that happened to be subcategories of the broader EF concept, the result of the subcategories was included in the core EF of the meta-analysis (e.g., task-switching is the subcategory of the broader concept of cognitive flexibility). After conducting a holistic meta-analysis for EFs, subgroup analyses were completed based on the specific and core EF domains: inhibitory control, working memory, and cognitive flexibility. Hedges’ *g* was preferred over Cohen’s *d* because its effect size index addresses bias in small sample sizes [52] and ideally incorporates a smaller number of studies (k < 20) [6]. The standardised mean differences (*g*) were calculated and weighted through inverse variance, thereby accounting for respective sample sizes, varying outcomes, and cognitive measures. The magnitude of Hedges’ *g* values was interpreted as small (< 0.2), moderate (0.5), and large (> 0.8) effect size [53]. Further, a random-effects model enabled the heterogeneous distribution of effect size [6]. The statistical heterogeneity was assessed (*I*^*2*^) with a *p*-value calculated for *Q* statistics. Specifically, *I*^*2*^ values signified whether heterogeneity was small (≤25%), medium (50%), or large (≥75%) [54].

The cut-off point was set at 50% of *I*^*2*^ value to estimate the heterogeneity of included studies; *I*^*2*^ statistics value of greater than 50% indicated heterogeneity. Furthermore, the sensitivity analysis (i.e., one study removed) was used to inspect the impact of retention/removal of outliers and their influence on the overall effect size [6]. Outliers were assumed if the results remained significant (*p* < 0.05) and within the 95% confidence interval. The potential publication bias to determine the balance of the funnel plot for EFs was presented through a funnel plot calculating the standard error (y-axis) and effect size (x-axis). Also, a “Trim & Fill” method (i.e., random-effects model) was used to estimate the publication-bias-adjusted true effect size and the number of studies needed to balance the plot [55]. A statistical significance of *p* < 0.05 was set for all tests.

### Quality assessment

The Physiotherapy Evidence Database (PEDro) scale [56] was used to assess the methodological quality of the included studies. The PEDro scale is a reliable and valid instrument for evaluating the methodological quality of studies gauging the effects of exercise on cognitive functions. It is used in both randomised control trials (RCTs) and non-RCTs for children with Autism spectrum disorder (ASD) [57] as well as the general population [20]. The PEDro scale has 11-item rating criteria for eligibility, randomisation, allocation, blinding (i.e., subjects and experimenter), intention-to-treat, between-group comparison, and point measures [58]. The PEDro scores range from 0 to 10, and the median score is 5. Notably, a prior review determined that blinding might be impossible in some cases. In many exercise intervention trials, it is rare to witness two points being allocated for blinded participants and blinded therapists [59]. Considering this limitation, the scoring system was divided into three categories: high quality ≥6, moderate quality = 4–5, and low quality ≤3 [57]. Moreover, two reviewers independently evaluated the methodological quality of the included studies based on the rating criteria [58] and calculated the overall study quality. Discrepancies regarding quality ratings were deliberated until consensus was reached. If an agreement could not be reached between the two reviewers, a third researcher made the final call.

## Results

### Descriptive characteristics of included studies

Overall, 21 studies [28, 34, 50, 60–77] were included in the systematic review and the characteristics of each study are summarised in Table 1. All finalised studies were undertaken between 2011 and 2020. In terms of geographic location, 10 studies were conducted in Asia (six in Taiwan, three in Korea, and one in Iran), four studies in North America (three in the USA, one in Canada), six studies in Europe (four in Switzerland, two in Germany), and one study in South America (Brazil). Collectively, 12 studies adopted an RCT design, three studies employed the NRS, and six studies used a crossover or randomised crossover design. The total sample included 664 children and adolescents with ADHD with ages between 6 and 18 years. Altogether, 18 studies targeted participants with formal ADHD diagnoses, following the formal diagnostic criteria. Accordingly, the Diagnostic and Statistical Manual of Mental Disorders (DSM) (fourth or fifth edition) criteria were commonly utilised as the gold standard by clinicians or psychiatrists [78]. Overall, eight studies employed clinical settings, guided by experienced experimenters or coaches who were familiar with the treatment approach. Specifically, two studies were undertaken at schools (i.e., during the after-school time), four studies targeted the university gym, two studies were applied at home, and the last two interventions depended on community facilities, such as swimming pools. The exercise interventions were divided into two categories: AE and CEE. In general, 10 studies adopted CEE training (i.e., exergaming, table tennis, and basketball), nine studies selected AE as a treatment approach (i.e., running, cycling, and swimming), and two studies used mixed training procedures comprising both AE and CEE. The intervention intensity ranged from light to vigorous, and seven studies adopted a moderate-to-vigorous physical activity (MVPA) level. The frequency of the intervention ranged from one to six times per week, with each session lasting 5 to 90 min. Furthermore, nine studies focused on acute exercise intervention with a duration ranging from 5 to 30 min, while 12 studies adopted a chronic exercise intervention ranging from 6 to 12 weeks, with a total duration of 720 to 4500 min. In addition, six studies implemented interventions for 6 to 8 weeks and another six studies intervened for 10 to 12 weeks. The effects of exercise interventions on core EFs were assessed by 21 studies, while six studies assessed working memory, 15 examined inhibitory control, and 11 targeted cognitive flexibility. Compared to studies focusing on the general population [15, 16], few studies described the results of higher-level EFs. Moreover, seven neurocognitive tasks were frequently used by researchers to assess EFs in children and adolescents with ADHD. Further, the Tower of London [79] and Digit Span Forward and Backward Test [80] were commonly used to measure working memory, and the Go-No-Go Task [81], Flanker Task [82], and Stroop Task [83] were frequently employed to evaluate inhibitory control. Lastly, the Trail Making Task [84] and Wisconsin Card Sorting Test [85] were selected to measure cognitive flexibility (see Supplementary Table for review).

**Table 1:** Descriptive characteristics of included studies

Study Name (Year, Country/ Region)	Research Design	Participant Characteristics			Intervention Components							Outcome Measures	Main Findings
		Participants (Age Range; Gender-M%; Diagnostic Methods)	Age (Control)	Sample Size (IG/CG)	Exercise Sessions	Setting	Program (Control)	Type	Intensity	Frequency	Length (mins)		
Kang et al.^a^ (2011, Korea) [77]	RCT	7–12; M-100%; K-ARS-PT	8.4 ± 0.9 (8.6 ± 1.2)	28 (15/13)	Chronic	NR	Sports Therapy (Education)	CEE	NR	90-min/session, twice/wk	6 wk. (1080)	• *WM*: Digit Symbol Test • CF: TMT	• *WM*+(*p* = 0.02) • CF+(*p* = 0.04)
Chang et al.^a^ (2012, Taiwan) [28]	RCT	8–13; M-93%; DSM-4	10.45 ± 0.95 (10.42 ± 0.87)	40 (20/20)	Acute	Clinical	Running (Watching video)	AE	Moderate	30-min/session/	1 session (30)	• *IC*: Stroop • CF: WCST	• *IC*+(*p* < 0.01) • CF+(*p* < 0.05)
Verret et al.^a^ (2012, Canada) [69]	nRCT	7–12; M-90%; DSM-4	9.1 ± 1.1	21 (10/11)	Chronic	School	PA programs	CEE	MVPA (77% HRmax)	45-min/session, 3 times/wk	10 wk. (1350)	• *IC*: TEAC	• *IC*+(*p* = 0.05)
Gawrilow et al.^a^ (2013, Germany) [75]	RCT	8–13; M-100%; ICD-10	10.47 ± 1.49	47 (23/24)	Acute	Clinical	Trampoline (Coloring in pictures)	AE	Vigorous	5-min/session	1 session (5)	• *IC*: GNG	• *IC*+(*p* < 0.05)
Pontifex et al. (2013, USA) [67]	Crossover	8–10; M-70%, DSM-4	9.46 ± 0.4 (9.8 ± 0.1)	40(20/20^TD^)	Acute	Clinical	Running (Reading)	AE	Moderate (65–75% HRmax)	20-min/session	1 session (20)	• *IC*: Flanker task	• *IC*+(d = 0.09)
Chang et al.^a^ (2014, Taiwan) [72]	nRCT	5–10; M-85%; DSM-4	8.78 ± 8.33 (8.19 ± 7.65)	27 (14/13)	Chronic	Community	Aquatic exercise	AE	Moderate	90-min/session, twice/wk	8 wk. (1440)	• *IC*: GNG	• *IC*+(*p* = 0.004)
Choi et al.^a^ (2015, Korea) [73]	RCT	13–18; M-100%; DSM-4	15.8 ± 1.7 (16.0 ± 1.2)	30 (13/17)	Chronic	NR	Sports Therapy (Education)	CEE	Light (60% HRmax)	90-min/session, 3 times/wk	6 wk. (1620)	• CF: WCST	• CF+(*p* = 0.03)
Chuang et al. (2015, Taiwan) [74]	RCO	8–12; M-84%; Psychiatric physician	9.42 ± 1.38	19	Acute	Clinical	Running (Watching video)	AE	MVPA (60% HRR)	30-min/session	1 session (30)	• *IC*: GNG	• *IC*+(*p* = 0.0039) • shorter RT in Go • stimuli
Piepmeier et al. (2015, USA) [66]	RCO	8–13; M-63%; Single question	10.14 ± 1.96 (11.22 ± 2.43)	32 (14/18^TD^)	Acute	Clinical	Cycling (Watching video)	AE	Moderate	30-min/session	1 session (30)	• *IC*: Stroop • CF: *TMT* • *WM*: TOL	• *IC*+(*p* = 0.004) • *CF***00**(*p* > 0.05) • *WM***00**(*p* > 0.05)
Ziereis & Jansen^a^ (2015, Germany) [71]	RCT	7–12; M-74%; ICD-10	IG^1^ 9.2 ± 1.3 IG^2^ 9.6 ± 1.6 (9.5 ± 1.4)	39 (IG^1^12, IG^2^11/16)	Chronic	Gym	G^1^: aerobic & motor skills; IG^2^: aerobic	AE & CEE	NR	60-min/session/wk	12 wk. (720)	• *WM*: DSFBT; LNST	• *WM*+(*p* < 0.0 • measured by LNST
Bustamante et al.^a^ (2016, USA) [70]	RCT	6–12; M-69%: DSM-4	9.4 ± 2.2 (8.7 ± 2.0)	34 (18/16)	Chronic	School	Cooperative games & sports (art project)	CEE	Moderate (75% HRmax)	90-min/session, 5 times/wk	10 wk. (4500)	• *IC*: STOPIT • *WM*: AWMA	• *IC*+(d = 0.67) • *WM*+(d = 0.29)
Hung et al. (2016, Taiwan) [76]	Crossover	8–12; M-97%; ADHD test	10.24 ± 1.78 (10.20 ± 1.09)	34	Acute	Clinical	Running (Watching video)	AE	MVPA (50–70% HRR)	30-min/session	1 session (30)	• CF: Task Switching Paradigm	• CF+(*p* < 0.05) • shorter RT in global • switch costs
Memarmoghaddam et al.^a^ (2016, Iran) [64]	RCT	7–11; NR SNAP-4	8.31 ± 1.29 (8.29 ± 1.31)	36 (19/17)	Chronic	Gym	AE & goal-directed exercise	CEE	MVPA (65–80% HRR)	90-min/session, 3 times/wk	8 wk. (2160)	• CF: Stroop • *IC*: GNG	• CF+(*p* = 0.00) • *IC+*(*p* = 0.00)
Pan et al.^a^ (2016, Taiwan) [34]	RCT	6–12; M-100%; DSM-4	8.93 ± 1.49 (8.87 ± 1.56)	32 (16/16)	Chronic	Gym	Table tennis	CEE	NR	70-min/session, twice/wk	12 wk. (1680)	• *IC*: Stroop	• *IC*+(*p* < 0.01)
Lee et al.^a^ (2017, Korea) [61]	RCT	6–10; M-100% DSM-4	8.83 ± 0.98 (8.83 ± 0.98)	12 (6/6)	Chronic	NR	Jump rope & ball skills	CEE	MVPA (45–75% HRR)	60-min/session, 3 times/wk	12 wk. (2160)	• *IC*: Stroop	• *IC*+(*p* < 0.01)
Ludyga et al. (2017, Switzerland) [62]	Crossover	11–16; M-62%; DSM-4	12.8 ± 1.8 (13.5 ± 1.3)	34 (16/18^TD^)	Acute	Clinical	Cycling & Coordinative exercise (Watching video)	AE & CEE	Moderate (65–70% HRmax)	20-min/session	1 session (15)	• *IC*: Flanker task	• *IC*+(*p* < 0.001) • decrease of RT • following exercise
Benzing et al.^a^ (2018, Switzerland) [50]	RCT	8–12; M-83%; ICD-10	10.46 ± 1.35 (10.50 ± 1.41)	46 (24/22)	Acute	Home	Exergaming (Watching video)	CEE	MVPA	15-min/session	1 session (30)	• *WM*: CSB • *IC*: Flanker task • CF: Flanker task	• *WM***00**(*p* = 0.995) • *IC*+(*p* < 0.05) • CF*+*(*p* < 0.05)
Benzing & Schmidt^a^ (2019, Switzerland) [60]	RCT	8–12; 84%; ICD-10	10.46 ± 1.30 (10.39 ± 1.44)	51 (28/23)	Chronic	Home	Exergaming (Watching video)	CEE	MVPA	30-min/session, 3 times/wk	8 wk. (720)	• *WM*: CSB • *IC*: Simon task • CF: Flanker task	• *WM***00**(*p* = 0.482) • *IC*+(*p* < 0.049) • CF*+*(*p* < 0.029)
Pan et al.^a^ (2019, Taiwan) [65]	nRCT	7–12; M-100%; DSM-4	9.08 ± 1.43 (8.90 ± 1.66) TD 9.14 ± 1.54	60 (15^IG^/15^CG^/30^TD-CG^)	Chronic	Gym	Table tennis	CEE	NR	70-min/session, twice/wk	12 wk. (1680)	• *IC*: Stroop • CF: WCST	• *IC*+(*p* = 0.41) • CF+(*p* < 0.05)
Silva et al.^a^ (2019, Brazil) [68]	RCT	11–14, M-70%, DSM-4	12.0 ± 2.0 (12.0 ± 1.0)	20 (10/10)	Chronic	Community	Swimming	AE	NR	45-min/session, twice/wk	8 wk. (720)	• CF*:* The Test of Trails	• CF+(*p* = 0.042)
Ludyga et al. (2020, Switzerland) [63]	Crossover	11–16; M-65%; DSM-5	12.8 ± 1.8 (13.5 ± 1.3)	34 (16/18^TD^)	Acute	Clinical	Cycling (Watching video)	AE	Moderate (65–70% HRmax)	20-min/session	1 session (20)	• CF: The Alternate Uses Task	• CF+(*p* = 0.43) • higher task performance scores following exercise

*ACT* Assisted Cycling Therapy, *AE* aerobic exercise, *AWMA* Automated Working Memory Assessment System, *CBTT* Corsi block-tapping task, *CEE* cognitively engaging exercise, *CF* cognitive flexibility, *CG* control group, *CTT* Color Trails Test, *CSB* color span backwards, *DSFBT* Digit span forward and backward test, *DSM-4 and-5* Diagnostic and Statistical Manual of Mental Disorders, Fourth Edition and Fifth Edition, *GARS-2* the Gilliam Autism Rating Scale, 2nd edition, *GNG* Go-No-Go task, *IC* inhibitory control, *ICD-10* International Classification of Diseases, Tenth Revision, *IG* intervention group, *K-ARS-PT* Korean version of the parent and teacher version of DuPaul’s ADHD Rating Scale, *LNST* Letter–number-sequencing task, *NR* no report, *NYG* Nei Yang Gong, *PPT* pre-post-test, *RCO* Randomized crossover, *RCT* Randomized Control Trial, *SNAP-4* Swanson, Nolan and Pelham Rating Scale, Fourth Edition, *STOPIT* Stop-Signal Inhibition Task, *TD* typical developing children, *TEAC* Test of Everyday Attention for Children, *TMT* Trail Making Test, *TOL* Tower of London, *VC* Voluntary Cycling, *WCST* Wisconsin Card Sorting Test, *WM* working memory, *%HRmax* Percentage of maximal heart rate, *%HRR* Percentage of heart-rate reserve (i.e., the training intensity zone is based on the ACSM Guideline 2018)

^a^ Studies included in the meta-analysis; +: significant statistical improvement; 00: no statistically significant change

### Meta-analysis of effects of exercise interventions on overall and core EFs

**Fig. 2:**
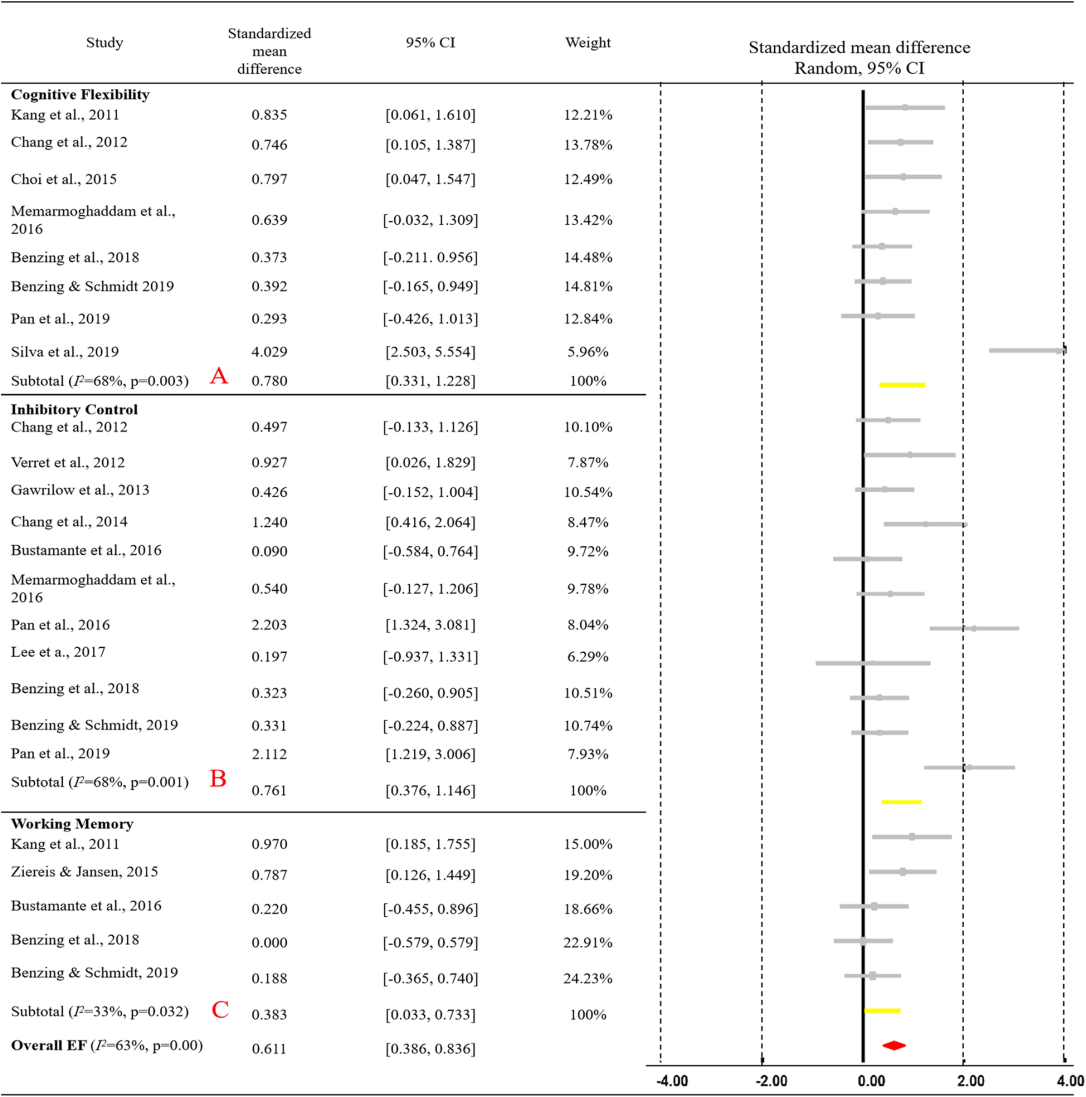
Forest plot for meta-analysis regarding the effect of exercise interventions on different EF domains

Among the 21 studies, 15 (12 RCT and 3 NRS) were identified as suitable for meta-analysis. Alternatively, 15 studies constituting data of 493 ADHD individuals were integrated into the meta-analysis. Results are exhibited in Fig. 2, indicating the effect size for ADHD (combined) on overall EFs. Additionally, a positive moderate-to-large (*SMD =* 0.611) training effect on overall EFs among children and adolescents with ADHD (95% CI [0.386 to 0.836], *p* < 0.01) and medium heterogeneity (*Q* = 62.200, *I*^*2*^ = 63%, *p* < 0.01) was evident. The EF tasks from the 15 ADHD studies were integrated through three core EF domains**.** Notably, eight studies highlighted significant moderate-to-large training effects (*g* = 0.780, 95% CI [0.331 to 1.228], *p* < 0.001) on cognitive flexibility (see Fig. 2a), with medium heterogeneity (Q = 21.936, *I*^2^ = 68%, *p* = 0.003), and 11 studies concentrating on inhibitory control (see Fig. 2b) specified significant moderate-to-large effects (*g* = 0.761, 95% CI [0.376 to 1.146], *p* < 0.01) together with medium heterogeneity (Q = 31.284, *I*^*2*^ = 68%, *p* = 0.001). Lastly, five studies on working memory (see Fig. 2c) revealed minimal-to-moderate significant effects of exercise interventions (*g* = 0.383, 95% CI [0.033 to 0.733], *p* < 0.05) in tandem with minimal heterogeneity (Q = 5.948, *I*^*2*^ = 33%, *p* = 0.203).

### Moderator analysis

To investigate the potential moderating effects, meta-regression was performed based on continuous variables, including age and intervention duration, while subgroup analyses were performed based on exercise type (AE or CEE), intervention intensity, and sessions of exercise (Acute or Chronic). There was a significant moderate-to-large effect (*SMD* = 0.611, 95% CI [0.386 to 0.836], *p* < 0.01) on overall EFs in children and adolescents with ADHD in tandem with medium heterogeneity (Q = 32.232, *I*^2^ = 63%, *p* < 0.01). As such, the higher heterogeneity for overall EFs implies that a moderator analysis could be performed to decipher the variability.

Table 2 recapitulates the results of subgroup analysis and meta-regression for overall EFs. The results of the former state that the effect of an exercise intervention on overall EFs was significantly moderated by intervention intensity and exercise sessions of intervention but not by exercise type. The moderate physical activity (MPA) (*g* = 0.539, 95% CI [0.29 to 1.048]) produced significant moderate-to-large training effects for overall EFs when compared to light physical activity (LPA) (*g* = 0.797, 95% CI [− 0.381 to 1.975]), MVPA (*g* = 0.349, 95% CI [− 0.080 to 0.778]) and vigorous physical activity (VPA) (g = 1.426, 95% CI [− 0.651 to 1.503]). As for the sessions of exercise of intervention, chronic exercise intervention produced significant moderate-to-large training effects (g = 0.789, 95% CI [0.513 to 1.065]) than acute exercise (g = 0.389, 95% CI [− 0.048 to 0.826]) on overall EFs in children and adolescents with ADHD. In the meta-regression, the duration of exercise and age were not found to have moderating effects on exercise or overall EFs.

**Table 2:** Moderator analysis of exercise intervention and overall EFs

**Categorical moderator**	Level	Standardized mean difference	95% CI	Test of heterogeneity		
				Q	d.f.	*P*-value
Intervention type	AE	1.001	[0.466, 1.536]	1.750	1	0.186
	CEE	0. 598	[0.335, 0.862]			
Intervention intensity	LPA	0.797	[−0.381, 1.975]	6.086	4	0.193
	MPA	0.539	[0.029, 1.048]			
	MVPA	0.349	[−0.080, 0.778]			
	VPA	1.426	[−0.651, 1.503]			
	Unclear	1.029	[0.650, 1.409]			
Exercise sessions of intervention	Acute	0.389	[−0.048, 0.826]	2.301	1	0.129
	Chronic	0.789	[0.513, 1.065]			
**Continuous moderator**	Level	β	95% CI	Q	d.f.	*P*-value
Age	6–18	−0.325	[−0.129, −0.064]	0.432	1	0.511
Duration of exercise (min)	5–4500	−0.0000	[−0.00011, 0.00011]	0.00014	1	0.990

*AE* aerobic exercise, *CEE* cognitively engaging exercise, *LPA* light physical activity, *MPA* moderate physical activity, *MVPA* moderate to vigorous physical activity, *VPA* vigorous physical activity

### Sensitivity analysis and publication bias

**Fig. 3:**
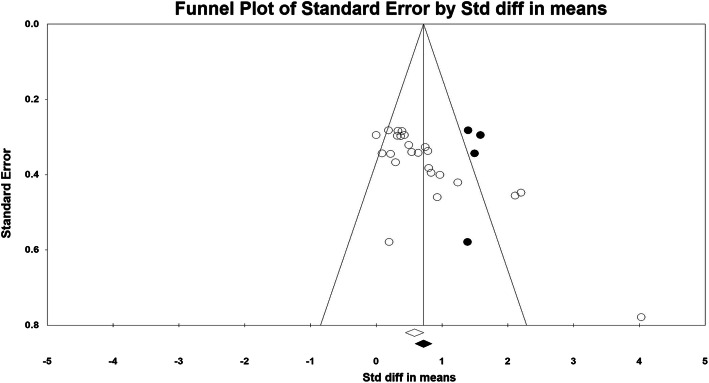
Funnel plot for visual inspection of publication bias

Specifically, two studies [64, 67] related to children with ADHD were found to be outliers (z = 4.915; z = 5.17, respectively), thus a “one study removed” test was performed. The single effect size score specified a change of − 0.109 [64] and − 0.129 [67], respectively, but remained significant (*p* < .01) and within the 95% confidence interval. Hence, the two outliers were retained. The funnel plot is presented in Fig. 3**,** and a “Trim & Fill” method was used to correct publication bias (i.e., *g* publication-bias-adjusted = 0.801, CI [0.565 to 1.037]); four studies were needed to balance the plot. The results indicated potential publication bias [55] in our included studies.

### Quality assessment of eligible studies

Table 3 depicts the quality assessment for the included studies. All studies fulfilled at least five criteria and most of them were RCTs. Hence, the resulting quality was high, with a mean score of 7. All incorporated studies had clear recruitment criteria and maintained a high retention rate during the intervention. The intention-to-treat analysis further demonstrated that the participants’ data were analysed according to their original assignment. However, only a few studies successfully blinded participants and therapists, due to the challenges associated with executing double-blind procedures in nonpharmacological studies.

**Table 3:** Methodological quality assessment for included studies

Study (Year, Country/Region)	Eligibility Criteria	Random Allocation	Concealed Allocation	Similar at Baseline	Subject Blinded	Therapist Blinded	Assessor Blinded	Dropout Rate	Intention-to-treat Analysis	Between-group Comparison	Points Measures	Total Score	Overall Study Quality
Kang et al.^a^ (2011, Korea) [77]	1	1	0	1	0	0	0	1	1	1	1	7	High
Chang et al.^a^ (2012, Taiwan) [28]	1	1	0	1	0	0	0	1	1	1	1	7	High
Verret et al.^a^ (2012, Canada) [69]	1	0	0	1	0	0	0	1	1	1	1	6	Moderate
Gawrilow et al.^a^ (2013, Germany) [75]	1	1	0	1	0	0	0	1	1	1	1	7	High
Pontifex et al. (2013, USA) [67]	1	0	0	1	0	0	0	1	1	1	1	6	Moderate
Chang et al.^a^ (2014, Taiwan) [72]	1	0	0	1	0	0	0	1	1	1	1	6	Moderate
Choi et al.^a^ (2015, Korea) [73]	1	1	0	1	0	0	0	1	1	1	1	7	High
Chuang et al. (2015, Taiwan) [74]	1	1	0	1	1	0	0	1	1	1	1	8	High
Piepmeier et al. (2015, USA) [66]	1	1	0	1	0	0	0	1	1	1	1	7	High
Ziereis & Jansen^a^ (2015, Germany) [71]	1	1	1	1	0	0	0	1	1	1	1	8	High
Bustamante et al.^a^ (2016, USA) [70]	1	1	1	1	0	0	1	1	1	1	1	9	High
Hung et al. (2016, Taiwan) [76]	1	0	0	1	0	0	0	1	1	1	1	6	Moderate
Memarmoghaddam et al.^a^ (2016, Iran) [64]	1	1	0	1	0	0	0	1	1	1	1	7	High
Pan et al.^a^ (2016, Taiwan) [34]	1	1	0	1	1	0	0	1	1	1	1	8	High
Lee et al.^a^ (2017, Korea) [61]	1	1	1	1	0	0	0	1	1	1	1	8	High
Ludyga et al. (2017, Switzerland) [62]	1	0	0	1	0	0	0	1	1	1	1	6	Moderate
Benzing et al.^a^ (2018, Switzerland) [50]	1	1	1	1	0	0	0	1	1	1	1	8	High
Benzing & Schmidt^a^ (2019, Switzerland) [60]	1	1	1	1	0	0	0	1	1	1	1	8	High
Pan et al.^a^ (2019, Taiwan) [65]	1	0	0	1	0	0	0	1	1	1	1	6	Moderate
Silva et al.^a^ (2019, Brazil) [68]	1	1	0	1	0	0	0	0	1	1	1	6	Moderate
Ludyga et al. (2020, Switzerland) [63]	1	0	0	1	0	0	0	1	1	1	1	6	Moderate

^a^ Yes = 1; No = 0

## Discussion

The current review investigated the impact of exercise interventions on EFs of children and adolescents with ADHD based on the outcome data of 15 studies. The meta-analysis revealed that exercise intervention has a positive moderate-to-large effect on overall EFs, inhibitory control, and cognitive flexibility in children and adolescents with ADHD. Further, the effects of exercise interventions on overall EFs were moderated by intervention intensity and exercise sessions of intervention.

Our findings of the positive effects of exercise interventions on overall EFs extend the results of previous systematic reviews. Beneficial relationships between exercise and cognition were determined in special populations, including children with mental impairment (ES = 0.43), physical disabilities (ES = 0.40) [39] and among children with neurodevelopmental disorders (*g* = 0.4, ASD, ADHD, and Developmental Coordination Disorder) [45]. Our meta-analysis expands the body of evidence related to the effects of physical exercise on cognition in individuals with ADHD [48]. We mentioned previously that the results of Tan’s review [48] included studies focusing on college students with ADHD, thus the effects of exercise on EFs of children and adolescents with ADHD cannot be generalised. Our review confirmed a moderate-to-large training effect (*g* = 0.611) in children and adolescents with ADHD. The underlying mechanism of exercise-induced EF improvements might be related to two aspects. First, from a practical perspective, children with special educational needs and initially lagging EFs gain more cognitive benefits from interventions than the general population [25]. Individuals with the poorest performance at the baseline have the greatest opportunity for improvement, whereas individuals with high levels of performance are limited in gaining more [86]. Another possible explanation is that EFs and prefrontal cortex are the first to appear in a child’s life [14]. However, delayed maturation of the frontal cortex, reduced cerebellum and prefrontal activity have been described in children with ADHD, resulting in EF deficiency [87]. Exercise boosts the co-activation between the cerebellum and the dorsolateral prefrontal cortex, wherein the activity level of the cerebellum is positively related to the dorsolateral prefrontal cortex [88]. In addition, children with these inherent impairments may experience pronounced EF improvements after exercise interventions; hence, more exercise interventions focusing on EFs are needed for children with ADHD.

Consistent with previous reviews, our review found similar positive effects in inhibitory control and working memory, consistent with previous reviews on the general population [15, 16] and individuals with ADHD [48]. Regarding cognitive flexibility, a moderate-to-large significant effect (*g* = 0.780) was confirmed in our meta-analysis. Nevertheless, this finding diverged from previous reviews that indicate a small but non-significant effect on the general population [15, 16] and individuals with ADHD [48]. Contrarily, our results affirmed the effects of exercise on the treatment of cognitive flexibility in children and adolescents with ADHD. This outcome may be because Tan’s review [48] only comprised five studies emphasising cognitive flexibility, with limited participants from a wide age range (7–25 years). Meanwhile, previous studies stated that cognitive flexibility was sensitive to AE during the formative years [62] and improved through child development but declined with age [14]. Thus, our review included more studies and focused on children and adolescents with ADHD to determine effects of exercise interventions on cognitive flexibility. We noticed that experimental studies examining cognitive flexibility were limited [89]. Cognitive inflexibility is generally reported in individuals with ADHD [28, 62], and this specific EF domain is related to learning and academic readiness; it predicts social understanding from middle childhood [90]. Therefore, development of cognitive flexibility should be advocated to parents and guardians.

Our review established that exercise intervention produced moderate-to-large training effects on overall EFs of children and adolescents with ADHD. This suggests that the effects of exercise training on the development of EFs in children and adolescents with ADHD appear to be greater than the effects on young adults (d = 0.55) [38]. EFs are essential for youth development, regulating daily behaviours and emotions in social, academic, and athletic settings [15]. Nonetheless, children with ADHD demonstrate cognitive deficits [91]. Our results corroborated that childhood is a sensitive period for EF development [14] and exercise interventions are beneficial for EF development and the wellbeing of young people [13, 37].

Our review identified intervention intensity as a moderator, and the benefits of exercise interventions were observed in the dose of MPA rather than LPA, MVPA and VPA. One previous review stated that MPA was the most registered dose in the modulation of cognitive and brain health [37] and was regarded as the appropriate intensity for children with ADHD [72]. However, we lacked sufficient data to precisely account the optimal parameters of exercise interventions on EFs of children and adolescents with ADHD. Contrarily, previous reviews have summarised that an inverted-U effect with prolonged MVPA and MPA exemplifies a greater effect on cognition than light and vigorous-intensity exercises [20, 37].

Furthermore, we found that with exercise sessions of intervention as a moderator, greater training effects were identified through chronic exercise (g = 0. 983) than acute exercise (g = 0.415) in children and adolescents with ADHD. Meanwhile, three recent reviews validated a small positive effect of chronic exercise programmes on overall EFs of the general population, including children aged 3–7 years (g = 0.35) [92], preadolescent children aged 6–12 years (g = 0.24) [20] and children and adolescents aged 6–17 years (d = 0.20) [16]. Interestingly, the beneficial effects of chronic exercise intervention appear more robust in ADHD than in the general population. Additionally, our review indicated that longer duration of exercise was not related to better EFs of children and adolescents with ADHD. This fact may be due to the characteristics of ADHD and the core symptoms that children usually display (i.e., attention problems and difficulty focusing on one activity for a long period). Hence, targeted research is required to investigate the influence of intervention intensity, duration, and frequency to realise greater cognitive benefits.

Moreover, intervention type was seen to be a cardinal factor along with other potential moderators. Previous studies justified that mixed exercises, including cognitive training, were more likely to improve the symptoms of ADHD than aerobic exercise [33] and yielded beneficial training effects on children’s EFs [93]. Yet, our results could not support this view as we found that both CEE (exergaming, ball games, and Chinese mind-body exercises) and AE (swimming and jumping) had significant moderate-to-large effects on EFs of children and adolescents with ADHD. Past research also concurs that CEE has greater inhibition-related benefits for overweight children than typical physical education classes have for lean children [94]. In our review, children with ADHD improved their EFs regardless of the type of exercise intervention, which was consistent with previous studies specifying that any type of exercise intervention can facilitate the performance of EFs [40]. Nevertheless, due to the limited number of studies using AE interventions, it is difficult to distinguish the effects of AE and CEE for children and adolescents with ADHD. Future studies should explore the effects of various types of exercise on neuroplasticity and their correlation with EFs in children with ADHD.

Remarkably, only four studies were developed in school settings, however improvements in some aspects of behavioural and cognitive performance in children with ADHD were found [68, 71]. School settings are regarded as a primary institution for children with special educational needs to acquire health-promoting physical activity [95]. Previous studies showed that typical physical education classes resulted in cognitive improvement in children [13]. Therefore, tailor-made in-school exercise interventions, including physical education classes, should be provided for children and adolescents with ADHD. Meanwhile, the positive effects of exergaming were found in our review, as the sample size and number of studies were comparatively small, which limited the generalizability of the results.

To the best of our knowledge, this study is the first to review the effects of exercise interventions on EFs of children and adolescents with ADHD. Overall, the positive effects of exercise interventions on EFs were established in these special populations. Still, there are several limitations to this study. First, a limited number of studies and sample sizes were included, which makes it difficult to give a robust conclusion other than exercises having a positive and significant effect on EFs of children and adolescents with ADHD. Second, only one study focused on adolescents with ADHD [73] and three studies combined children and adolescents together [28, 67, 75], thus it was not possible to determine separate effect sizes. More research is needed to determine difference in the effects between children and adolescents with ADHD. Third, the assessment of cognitive tasks was inconsistent, which may distort the synthesisation of results due to high heterogeneity. Fourth, the majority of included participants were male; therefore, we could not examine gender differences. Lastly, because a limited number of studies were involved in this review, other higher-level EFs (e.g., planning) and potential moderators could not be identified.

## Conclusion

This review showed a moderate-to-large positive effect of exercise interventions on overall EF in children and adolescents with ADHD. Both aerobic exercise and cognitively-engaging-exercise showed positive effects on children and adolescents with ADHD. Well-designed chronic exercise interventions with MPAs may offer a promising avenue to improve multiple EFs of children and adolescents with ADHD, especially concerning their inhibitory control and cognitive flexibility.

## Supplementary Information

**Additional file 1.**

**Additional file 2.**

## Data Availability

All data generated or analysed during this study are included in this article.
