# Supplementary material for: The impact of exercise interventions concerning executive functions of children and adolescents with attention-deficit/hyperactive disorder: a systematic review and meta-analysis
Source: Int J Behav Nutr Phys Act. 2021 May 22;18:68. doi: 10.1186/s12966-021-01135-6 (PMC8141166; doi:10.1186/s12966-021-01135-6)
Supplement: Supplementary file 1 — Additional file 1. [file 12966_2021_1135_MOESM1_ESM.docx]

*Supplementary Table. The core and higher-order executive functions*

| Domains of Executive Functions | Definition | Examples of Neurocognitive Tasks |
| --- | --- | --- |
| Core Executive Functions | | |
| Cognitive Flexibility | Changing perspectives or approaches to a problem, flexibly adjusting to new demands, rules, or priorities (as in switching between tasks) [14] (p. 137) | Wisconsin card sorting sask [76], Dimensional card sorting test [89] |
| Inhibitory Control | Being able to control one’s attention, behavior, thoughts, and/or emotions to override a strong internal predisposition or external lure, and instead do what’s more appropriate or needed. [14] (p. 137) | Stroop task [74], Simon task [90], Flanker task [73], Go/no-go task [72] |
| Working Memory | Holding information in mind and mentally working with it (or said differently, working with information no longer perceptually present) [14] (p. 137) | Digit span forward and backward test [71], Corsi block test [71], N-back tasks [91] |
| Higher-order Executive Functions | | |
| Planning | Before embarking on an action sequence which is novel or complex, we usually spend some time thinking what we are about to do, how best to achieve the goal in what order to perform the individual actions, and how much time and effort will need to be allocated to the task [70] (p. 49) | Tower of London [70]  Tower of Hanoi [70] |
| Reasoning | Ability to draw relationships between disparate or dissimilar phenomena [92] (p. 87) | Cattell culture fair test [93] Test of strategic learning [94] |
| Problem-solving | A person is confronted with a problem when he/she wants something and does not know immediately what series of actions he can perform to get it [95] (p. 200) | Tower of London [70],  Dog-cat-mouse task [96] |
