# Supplementary material for: The impact of exercise interventions concerning executive functions of children and adolescents with attention-deficit/hyperactive disorder: a systematic review and meta-analysis
Source: Int J Behav Nutr Phys Act. 2021 May 22;18:68. doi: 10.1186/s12966-021-01135-6 (PMC8141166; doi:10.1186/s12966-021-01135-6)
Supplement: Supplementary file 2 — Additional file 2. [file 12966_2021_1135_MOESM2_ESM.pdf]

Database	Search strategy	Limits
CINAHL Complete; MEDLINE; Web of Science; Eric; PsychINFO, SPORTDiscus with Full Text,	(physical activit* OR physical fitness OR physical exercise OR physical education OR leisure activit* OR motor activit* OR sport* participation OR fitness OR cardiovascular fitness OR exercis* OR acute exercise OR chronic exercise OR healthy exercise OR aerobic exercise OR resistance exercise OR anaerobic exercise OR intervention OR baseball OR basketball OR bicycling OR boxing OR football OR golf OR gymnastics OR hockey OR Tai Ji OR mountaineering OR racquet sports OR tennis OR skating OR snow sports Skiing OR soccer OR sports for persons with disabilities OR track and field OR youth sports OR wrestling OR weight Lifting OR games recreational OR motor skills OR motor intervention OR braces OR motor learning OR behavior skills training OR task specific training OR weight bearing exercise OR strength training OR aerobic training OR running OR agility OR swimming OR aquatic exercise OR horse riding OR trampoline OR snowshoeing OR skating OR exergaming OR skateboarding OR dance OR walking OR treadmill) AND (executive function OR executive dysfunction OR working memory OR problem-solving OR decision making OR planning OR inhibitory control OR response inhibition OR inhibit* OR shifting OR switching OR neuropsychologic* OR cognitive control OR set-shifting OR cognitive flexibility OR impulse control OR attention OR cogniti* OR mental flexibility OR mental set shifting OR self-control OR behavioral inhibition OR interference control OR reasoning) AND (children OR childhood OR school-age OR youth OR adolescents OR teenagers OR students) AND (ADHD OR ADD OR attention-deficit disorder* OR attention-deficit hyperactivity disorder*) [Title]	-Human;<br>-English language
